# Utilizing digital predictive biomarkers to identify Veteran suicide risk

**DOI:** 10.3389/fdgth.2022.913590

**Published:** 2022-10-18

**Authors:** Jackson G. Holmgren, Adelene Morrow, Ali K. Coffee, Paige M. Nahod, Samantha H. Santora, Brian Schwartz, Regan A. Stiegmann, Cole A. Zanetti

**Affiliations:** ^1^ Rocky Vista University College of Osteopathic Medicine, Ivins, UT, United States; ^2^ Rocky Vista University College of Osteopathic Medicine, Parker, CO, United States; ^3^ Department of Medical Humanities, Rocky Vista University College of Osteopathic Medicine, Parker, CO, United States; ^4^ Department of Tracks and Special Programs, Rocky Vista University College of Osteopathic Medicine, Parker, CO, United States; ^5^ Flight Medicine, US Air Force Academy, Colorado Springs, CO, United States; ^6^ Chief Health Informatics Officer, Ralph H Johnson VA Health System, Charleston, SC, United States

**Keywords:** digital predictive biomakers, veterans, veteran suicide, digital health, biomarkers

## Abstract

Veteran suicide is one of the most complex and pressing health issues in the United States. According to the 2020 National Veteran Suicide Prevention Annual Report, since 2018 an average of 17.2 Veterans died by suicide each day. Veteran suicide risk screening is currently limited to suicide hotlines, patient reporting, patient visits, and family or friend reporting. As a result of these limitations, innovative approaches in suicide screening are increasingly garnering attention. An essential feature of these innovative methods includes better incorporation of risk factors that might indicate higher risk for tracking suicidal ideation based on personal behavior. Digital technologies create a means through which measuring these risk factors more reliably, with higher fidelity, and more frequently throughout daily life is possible, with the capacity to identify potentially telling behavior patterns. In this review, digital predictive biomarkers are discussed as they pertain to suicide risk, such as sleep vital signs, sleep disturbance, sleep quality, and speech pattern recognition. Various digital predictive biomarkers are reviewed and evaluated as well as their potential utility in predicting and diagnosing Veteran suicidal ideation in real time. In the future, these digital biomarkers could be combined to generate further suicide screening for diagnosis and severity assessments, allowing healthcare providers and healthcare teams to intervene more optimally.

## Introduction

In the United States, Veteran suicide rates have been steadily rising since 2009 (1). According to the 2020 National Veteran Suicide Prevention Annual Report, since 2018 an average of 17.2 Veterans have died by suicide each day (2). There is a significant demand to redefine how suicidality is effectively identified and triaged to more readily identify patterns that correlate to suicidal ideation. Further exacerbating this current medical predicament, the COVID-19 pandemic has only served to further highlight the significant shortage of mental health professionals in the United States (3, 4). In order to combat the rising rates of suicide, it is paramount to more optimally identify Veterans with elevated suicide risk. This approach has been supported by the White House as part of the five priorities for reducing military and Veteran suicide (5).

### Risk factors for suicide within the Veteran population

In the effort to lower Veteran suicide rates, many risk factors for increased suicide risk have been identified (6). Several of the strongest predictors being attempt history, post-traumatic stress disorder (PTSD) symptoms, alcohol use disorder, depression, and anxiety (7, 8). Social factors such as local unemployment and community engagement have also be described as relating to Veteran suicide risk (9). Those with multiple risk factors were shown to have even higher suicide risk (8). Many biomarkers have also been related to suicide risk in the Veteran population (10). Recently, structural brain imaging findings, neurotransmitter levels, the hypothalamic pituitary adrenal axis, inflammatory response, lipid levels, and neuroplasticity have all been implicated with risk of suicide in the general population (11). Additionally, factors such as heart rate variability, sleep disturbances, and speech patterns have also been correlated with suicide risk (12–14). Identifying Veterans with biomarkers such as these could lead to more accurate suicide screening (15).

### Suicide screening and clinical tools

Screening refers to the systematic application of a test or enquiry to identify individuals at sufficient risk of a specific diagnosis to warrant further investigation (16). Currently, the screening of Veterans for suicide risk is limited to those who are willing to divulge their mental health status on self-report assessments, patient interviews in a clinical setting, suicide hotlines, and self, family, or friend reporting to a healthcare professional (17). In 2019, utilizing the VA's Suicide Risk Identification Strategy (Risk ID), the prevalence of suicidal ideation within the Veteran population was 3.5% (18). Risk ID is a suicide risk screening process that uses the nine item Patient Health Questionnaire (PHQ-9), Columbia-Suicide Severity Rating Scale (C-SSRS), and the VHA Comprehensive Suicide Risk Evaluation (CSRE) (18). Patients who screen positive *via* the PHQ-9 move on to the C-SSRS for further evaluation, and those who screen positive on the C-SSRS move onto the CRSE. The PHQ-9 is a tested and reliable measure of depression severity (19). Item 9 of the PHQ-9 assesses the presence of suicidal ideation within the past two weeks (18). Higher levels of suicidal ideation, as indicated by item 9, have been associated with a higher risk of suicide among Veterans (20). However, many Veteran suicides occur in patients who respond “not at all” to item 9 (20). The C-SSRS is a six-item screener designed to determine the presence of suicidal ideation, method, intent, and plan during the last thirty days (21). In 2011, this screener was suggested to be suitable for assessing suicidal ideation and behavior in adolescence and adults (21). The CSRE is a suicide screening tool developed by the Risk ID workgroup in 2019 (18). Interestingly, the CSRE is not a scripted assessment and encourages providers to use their clinical expertise and knowledge of the Veteran to stratify both acute and chronic suicide risk (18). Stratifying Veteran suicide risk based on severity and temporality has been shown to help providers create individualized treatment plans (22).

However, the PHQ-9 and C-SSRS have been found to be insufficient screening tools for suicide risk in at-risk populations, including the Veteran population (23, 24). Yarborough et al. found that among individuals with substance use disorders, in 46% of outpatient visits that were followed by a suicide attempt within 90 days, the patients did not meet the qualification criteria for having suicidal ideation utilizing these tools (24). Using this data, patients who are not identified as having suicide risk *via* the PHQ-9 should not be declared immune to subsequent suicide attempt or heightened risk of suicidality (24). Additionally, the C-SSRS has recently been shown to be insensitive to suicide risk thirty days after patients being discharge from the emergency department (ED) (23). Simpson et al. found that of the adult United States citizens that presented to the ED and subsequently died of suicide within the following twelve months, the majority did not receive ED psychiatric care and had a negative screening *via* the C-SSRS. Since these tools exhibit questionable results, it would be ideal for Veterans to be screened and strategically monitored for suicidal ideation as the ideation evolves (18). Some of the largest challenges when screening Veterans for suicide risk stem from ineffective screening tools that fail to provide patient insights in real-time (25). Incorporating digital biomarkers as part of an active screening process may help create the capability to help overcome these challenges.

### Smartphones and wearable technologies

As smartphone and wearable technologies are becoming more common in everyday life, they are also becoming more integrated into medical research and clinical medicine (26). This is partly due to mobile sensor technology being capable of measuring digital biomarkers outside of clinical visits (27). Mobile technologies have also been shown to encourage healthy lifestyles by providing real-time information to patients (28). The affordability of these technologies is also leading to their increased use in health monitoring (29). These technologies are currently being employed in many fields such as Alzheimer's treatment, cancer research, and mental health (30–32).

### Digital predictive biomarkers

As the medical world further navigates the evolution of digital medicine technologies, Torous et al. posits that mobile technology can aid in the early detection and reduction of suicide and reduction of suicide rates (33). Through the use of digital medicine technologies designed for tracking changes in mood, stress, anxiety, meditation, and coping, digital biomarkers can shed light on the overall health of an individual (34, 35). Utilizing smart phones, wearable technologies, and applications, healthcare providers can more strategically monitor these digital biomarkers for the early signs of depression, suicidal thoughts, and suicidal risk (34, 36). In order to best understand how to approach depression and suicidality from a digital health perspective, it is essential to first define the term “digital biomarker.” Digital biomarkers are metrics that are objectively gathered and measured by wearable, implantable, or ingestible devices and sensors that can be used as an indicator of normal or pathologic processes (37). This broad definition describes many types of biomarkers, including digital predictive biomarkers, which are specifically used to identify individuals who are more likely to experience an effect from exposure to a stimulus compared to a similar person without the biomarker (38). For example, a digital predictive biomarker might be overall daily activity levels as they relate to the onset of a disease process (39). Mobile technology can be utilized to perform real-time collection of relevant digital predictive biomarkers, which can lead to more timely interventions (40). The widespread availability of technologies such as smartphones and wearable devices have led to the opportunity to utilize digital predictive biomarkers to help better predict and potentially prevent suicide. Although screening for digital predictive biomarkers has been suggested in the realm of Veteran suicide prevention, no methods have been suggested for combining these risk factors with traditional screening tools to improve accuracy and efficiency of Veteran suicide risk screening.

There is a need to identify digital predictive biomarkers that correlate to increased risk of Veteran suicide, as well as biomarkers that can serve as early identifiers and proxy markers for behaviors indicative of suicidal ideation to better inform providers of Veteran mental health status in real time. Given the current limitations of the PHQ-9 and C-SSRS screening tools currently in clinical practice, there exist various opportunities to incorporate digital biomarkers into our existing screening modalities to further enhance sensitivity, specificity, and efficiency of suicidality detection. This review aims to provide examples of current digital predictive biomarkers that have already been correlated with Veteran suicide risk. Then, present a method of incorporating them into the screening process for Veteran suicide.

## Results

The following digital predictive biomarkers were chosen based on their levels of evidence, ability to be captured in real-time *via* smartphone or wearable technologies, and they had to be related to the Veteran's physiology. Biomarkers such as social media usage and geographic information were excluded.

### Biomarkers collected during sleep

**Figure 1:**
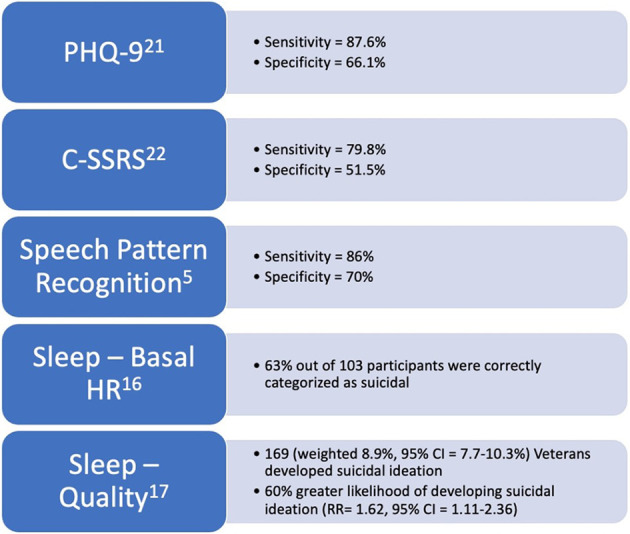
Figure stating the levels of evidence and statistical metrics of digital biomarkers and screening tools discussed in this review.

Lemogne et al. and Chang et al. described baseline heart rate collected through wearable devices to be a predictor of future suicide attempts (41, 42). These studies utilized electrocardiography (ECG) to measure resting heart rate (41, 42). ECG has also been utilized to establish links between suicide risk and heart rate variability, sinus arrhythmias, and QT variability (43–45). In 2019, Woodward et al hypothesized that sleep heart rate, which is a proxy for basal heart rate, i.e., resting heart rate, could differentiate Veterans with chronic PTSD who have suicidal ideations from those who do not (46). By analyzing all-night heart rates derived from pulse monitors, as well as snoring collected from another wearable device, it was found that sleep heart rate, snoring, and body mass index (BMI) were all able to differentiate between non-suicidal participants and ones with suicidal ideation. Sleep heart rate was shown to be significantly higher in participants identified as suicidal compared to those who were not. Snoring was also noted to be more prevalent. Finally, BMI was two points higher in participants coded as suicidal. Utilizing these digital biomarkers, 63% of participants were correctly categorized as suicidal or non-suicidal based on sleep heart rate, snoring, and BMI (Figure 1) (46). This data shows that the digital biomarkers sleep heart rate, snoring, and BMI may be used to identify Veterans with suicidal ideation. However, it is important to note that Lemogne et al. and Chang et al. lacked healthy control groups and were confined to patients without psychiatric comorbidities. Thus, comorbidities may play a larger role in the correlation between sleep vital signs and suicidal ideation (42).

### Sleep disturbance and quality

McCarthy et al., Bishop et al., and Don Richardson et al. discovered a significant association between sleep disturbances and suicidal ideation (47–49). Sleep disturbances include difficulty falling asleep and staying asleep, both of which qualify as metrics that can be monitored with digital technologies (47–49). Regressive analysis was used to investigate these associations while controlling for depressive, PTSD, and anxiety symptom severity (49). Significant associations between sleep disturbances and higher rates of suicidality were found to be linked by an increase in depressive symptom severity, ultimately contributing to the Veteran's suicidal ideations (49). Self-rated poor sleep quality was also correlated with a greater than 60% likelihood of a Veteran developing suicidal ideation (47). Another important consideration when determining how sleep disturbances may affect Veteran suicidality is the association between sleep disturbances and the timing of Veteran suicide events (50). When comparing the American Time Use Survey and the National Violent Death Reporting System, McCarthy et al. evaluated the association between suicide rates and nocturnal wakefulness. They found that a greater proportion of Veterans died by suicide between the hours of 00:00 and 03:00 than what would be expected based on the number of Veterans who were awake during that time frame. Thus, sleep disturbances may lead to a higher chance of death by suicide. The researchers further stratified the data into age brackets. They found that Veterans greater than 65 years of age are around ten times more likely to die of suicide between the hours of 00:00–03:00, while Veterans between the ages of 18–39 are at a six times higher risk before midnight. This data is useful because it could lead to more personalization in treatment plans. For instance, knowing that in older populations, observed suicides peak in the early morning could inform care givers to put in place strategic morning safety plans and interventions for Veterans older than 65, while evening and nighttime interventions may be more appropriate for younger Veterans between the ages of 18 and 39 (50). The authors make clear that while more research must be conducted to draw concrete associations between sleep disturbances and Veteran suicide, their data illustrates that using wearable devices to track sleep disturbances and sleep quality may lead to more personalized and appropriate intervention care plans for Veteran populations (50).

### Speech pattern recognition

Belouali et al. underscored how speech pattern recognition can be a digital biomarker for insight into suicidal ideation among Veterans (17). In this study, 124 Veteran participants provided 588 audio recordings of themselves answering opened ended questionnaires inquiring about their general health *via* a smartphone application that researchers designed. Recordings were self-submitted in a real-life setting and each recorded response included a PHQ-9. Using a machine-learning approach, an analysis was conducted on voice characteristics as well as textual features in transcribed audio. Veterans with suicidal ideation were found to have less animated, flatter voices with less vocal energy, more breathy voice quality, less abrupt changes, and increased monotony. These findings showed that audio collection using a mobile application outside of the clinic is useful in classifying suicidal ideation with comparable effectiveness to the traditional screening tools with 86% sensitivity and 70% specificity after analysis (Figure 1). Therefore, the use of speech pattern recognition technology may allow providers to intervene more strategically prior to a suicide attempt. Conversely, the authors noted that their collection techniques relied upon self-reporting, thus some of the recording could have been subjected to the Hawthorne effect and mislabeled if participants were not willing to divulge their suicidal ideation. However, when comparing their data, the researchers hypothesized that utilizing mobile applications encouraged patients to disclose more information compared to conventional screening techniques. The researchers also noted that demographics and mental states such as anxiety, depression, and PTSD might have confounded results. Further studies in this novel arena were recommended (17).

## Discussion

Increasing Veteran suicide rates is a national health crisis that deserves immediate attention (51). One approach to lowering suicide rates in the Veteran population could be to screen Veterans for suicide risk with digital predictive biomarkers, such as sleep vital signs, sleep disturbance, sleep quality, and speech pattern recognition. Improving accuracy and more strategic screening of Veterans is vital to identifying and triaging suicide risk to ensure proper care to those with the greatest need (18). Given that digital predictive biomarkers have been shown in previous research to correlate with the identification of increased suicidality, it may be beneficial for these biomarkers to be used as screening tools to assess suicide risk within the Veteran population. Current screening tools, such as the PHQ-9 and C-SSRS have been shown to be insufficient for identifying suicidal ideation. Na et al (2018) found that for the PHQ-9, suicidal ideation sensitivity and specificity was 87.6% and 66.1% respectively (Figure 1) (52). Furthermore, the C-SSRS was shown to have a sensitivity of 79.8% and a specificity of 51.5% (Figure 1) (53). Additionally, other suicide risk screening tools such as the Beck Scale for Suicidal Ideation, SAD PERSONS, Wald test, as well as the PHQ-9 and C-SSRS were shown to have poor predictive values for near-term events following ED admission (54). Interestingly, a systematic review of available suicide risk assessment tools found that no tool alone had sufficient accuracy to predict suicide (55). The tracking of digital predictive biomarkers could improving the chances of identifying suicide risk in Veterans.

### New method

Currently, screening for Veteran suicide risk is limited to suicide hotlines, patient reporting, patient visits, and family or friend reporting. By utilizing digital technologies, such as smartphones and wearables, digital predictive biomarkers could be continuously monitored outside of the healthcare setting. Increasing time sampling for suicidal ideation has been shown to be highly capable of detecting suicidal ideation at daily, and even hourly increments (56). Thus, continuous screening through the use of digital biomarkers could increase the probability of detecting suicide risk in previously undetected Veterans.

**Figure 2:**
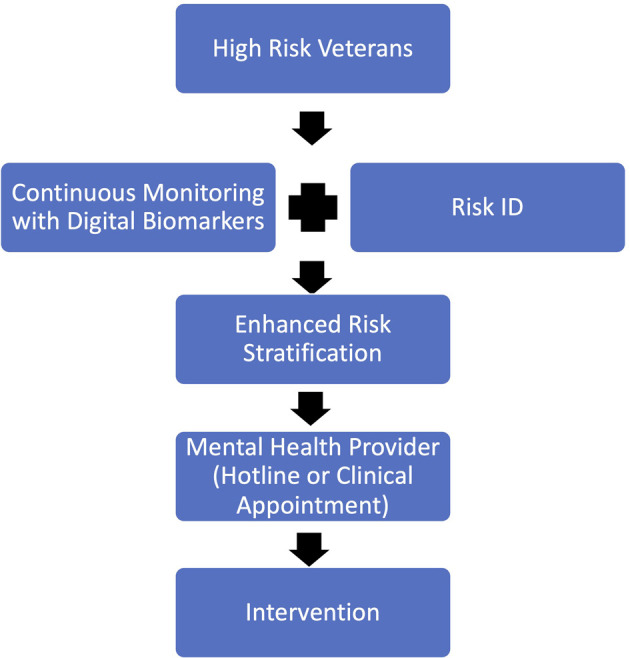
Proposed new method of screening Veterans for suicide risk which combines continuous monitoring via digital biomarkers with the current Risk ID process in order to enhance risk detection and stratification.

When determining who to screen with digital predictive biomarkers it is important to consider risk factors for suicide within the Veteran population. Veterans at high risk, such as those presenting with multiple risk factors, including but not limited to PTSD, depression, or previous attempt history should be screened utilizing digital biomarkers (Figure 2). Veterans at high suicide risk could stand to benefit the most from continuous monitoring because as stated earlier, suicidal ideation is a complex process that evolves in real-time (57). Once identified as high risk, Veterans would then have a mobile health application capable of safely and securely monitoring digital biomarkers installed onto their mobile device or current wearable technology. If the Veteran does not already possess such technology, a means of data collection, i.e., a mobile or wearable device must be provided for them.

Once a Veteran has screened positive for suicide risk utilizing digital predictive biomarkers, they must be put into contact with a provider that is able to create a personalized treatment plan quickly and effectively (Figure 2). Depending on the Veteran's location and proximity to resources the provider they are put in contact with could be through a hotline service, or an in-person clinical visit. The mobile monitoring technology could be configured in a way to send an alert the provider based on the Veterans preference. If the Veteran prefers to utilize hotline services, an alert would be sent to an available hotline representative to reach out to the Veteran in need. If the Veteran prefers to see an in-person provider, an alert would be sent to either their established mental health provider or the nearest VA health system where they can seek appropriate medical care. In either case, a provider would be notified and asked to reach out to the Veteran to start the treatment process as soon as possible.

Once the Veteran has been put into contact with the appropriate mental health provider, a personalized treatment plan must be developed and implemented (58). Tracking digital biomarkers could help develop these plans (59). As stated earlier, tracking digital biomarkers has been shown to accurately shed light on an individual's overall health as well as lead to more timely interventions. The data collected *via* mobile technologies must be made available to the providers creating treatment plans to enable them to create more personalized and effective treatment strategies for Veterans with suicide risk.

It is important to note that the proposed new method of screening Veterans for suicide risk should not replace the current Risk ID process, instead tracking digital biomarkers should augment the existing clinical screening process (Figure 2). There is a growing body of evidence that shows that suicide screening and evaluation in the clinical setting is effective, feasible, and can be completed without major interruptions to workflow (60). Thus, screening Veterans for suicide risk should continue in the clinical setting. However, since the current screening tools have been shown to be ineffective, screening could be augmented by tracking digital biomarkers outside of the clinical visit. Thus, increasing the chances of detecting Veteran suicide risk.

For these digital predictive biomarkers to be used effectively, Veterans with mental illness must be capable of interacting with a digital device with sustained longitudinal compliance. Meaning they must be capable and willing to consistently engage with a digital device to ensure effective screening and follow-up pending positive screening results. Studies have demonstrated that people with a mental illness were as likely as the general population to own a digital device with health apps installed (36). To address essential components of health equity, there would be a need to provide access to these technologies to Veterans who do not currently have access to compatible smartphones or devices. Additionally, it must also be determined if the Veteran population is willing to accept and engage with digital predictive biomarker technology. Betthauser et al. found the use of mobile applications to monitor behavioral health symptoms within the Veteran population feasible and accepted by the Veterans studied (61). This is encouraging data that shows if implemented, digital health technologies can be feasibly utilized within the Veteran population.

### Limitations

A limitation to the widespread implementation of digital predictive biomarkers in the context of suicide-prevention is that existing digital predictive biomarkers have not been thoroughly studied. Many are not clinically validated or developed specifically for the Veteran population and its particular risk factors. Also, data regarding the relationship between biomarkers like sleep vital signs, sleep disturbances, sleep quality, and speech pattern recognition, as to how they pertain to suicide risk, are limited and confounded for the Veteran population. Tucker et al. discovered that there are indirect effects of insomnia symptoms on suicidal ideation when participants subsequently experienced feelings of loneliness and lack of meaningful social relationships. This research did not find a direct association between insomnia symptoms and suicidal ideation alone (62). Further, it has been shown in prior literature that a lack of social relationships and an individual's belief that they are a liability to others, are associated with suicidal ideation across a variety of populations (63). This shows that the relationship between sleep and suicide is more complicated than previously thought (48, 64). However, Hilberg et al. identified sleep as a highly modifiable therapeutic target that can be used to target Veteran suicide prevention (65). This illustrates that even with the limited research in this topic, tracking sleep biomarkers shows great promise in potentially improving screening for Veteran suicide risk and subsequently aiding in the intervention of suicidal ideation.

To establish a truly objective measurement of each of these biomarkers, data must be generalizable across a wide range of studies (14). A standardized approach to data collection and collaboration with practicing physicians and healthcare providers to clinically validate the efficacy of these digital biomarkers is a logical next step towards research and implementation of digital predictive biomarkers into clinical practice (14). A safe and secure technology agnostic software solution that is focused on health maintenance and suicide prevention is warranted for this approach to be successful. In addition, there is a need for broad access to open-source data for continued research on this subject. The VA's use of synthetic data creates large stores of deidentified data that could be subsequently used for researching more effective screening methods.

As the medical field continues to work towards improving effective identification of suicide risk in the Veteran population, it becomes essential to balance early identification and the risk of overdiagnosis (66). If utilizing digital biomarkers were to improve screening sensitivity, specificity could be reduced, leading to is the potential for overdiagnosis in this area which needs to be considered and meaningfully addressed. Healthcare systems could be overwhelmed by the potential increase in Veterans identified as suicidal. Further research on the risk overdiagnosis is needed to balance the outcome of clinical benefit.

A major limitation within mental health treatment, as recently highlighted by the COVID-19 pandemic, is a significant shortage of mental health resources in the United States. Current recommendations from the United States Preventive Services Task Force recommends against screening for suicide if there is not adequate infrastructure to follow up with these high-risk individuals (67). While the proposed incorporation of digital biomarkers in conjunction with conventional screening tools will not directly provide additional healthcare infrastructure, it could enable providers to apply the limited resources more efficiently to Veterans with the greatest needs.

## Conclusion

The screening of Veterans for suicide risk requires careful and personalized assessments. Quantifiable digital biomarkers that correlate to increased risk of suicide can serve as early identifiers of potentially heightened suicidal ideation and may better inform providers of Veteran mental health status in real time. Given the limitations of the PHQ-9 and C-SSRS, there exists an opportunity to augment traditional screening tools with digital predictive biomarkers potentially creating more sensitive, specific, and efficient screening tools for Veteran suicide risk and prevention. Further studies are warranted to assess patient impact, data fidelity, and risk of overdiagnosis. In this review, sleep vital signs, sleep disturbance, sleep quality, and speech pattern recognition have been identified through preliminary research as potential data that correlates with suicidal ideation in the Veteran population. If these digital biomarkers were able to be utilized in conjunction with conventional screening tools, healthcare teams could better identify greater numbers of Veterans with acutely heightened suicide risk. By increasing real time awareness of suicidality and its predictive physiologic features, on-demand mental health services need to be more readily available to support Veterans. Thus, digital predictive biomarkers paired with conventional screening methods could help fulfill the White House's new strategy to reduce Veteran suicide by improving upon the current industry standard screening methods.

## References

[1] (NIMH) NIoMH. Suicide Online: National Institute of Mental Health (NIMH); 2022 [updated March 2022. Available from: https://www.nimh.nih.gov/health/statistics/suicide

[2] Affairs USDoV. 2021 National Veteran Suicide Prevention Annual Report. (2021).

[3] McGinty B. Medicare’s mental health coverage: How COVID-19 highlights gaps and opportunities for improvement. Issue Brief, New York, NY: The Commonwealth Fund (2020).

[4] Wisniewski H Gorrindo T Rauseo-Ricupero N Hilty D Torous J. The role of digital navigators in promoting clinical care and technology integration into practice. Digit Biomark. (2020) 4(Suppl. 1):119–35. 10.1159/000510144 33442585 PMC7768140

[5] Fact Sheet: New Strategy Outlines Five Priorities for Reducing Military and Veteran Suicide [press release]. (2021).

[6] Pfeiffer PN Ganoczy D Ilgen M Zivin K Valenstein M. Comorbid anxiety as a suicide risk factor among depressed Veterans. Depress Anxiety. (2009) 26(8):752–7. 10.1002/da.20583 19544314 PMC2935592

[7] van der Kolk BA van der Hart O Burbridge J. Approaches to the treatment of PTSD. (1995):421–43.

[8] Lee DJ Kearns JC Wisco BE Green JD Gradus JL Sloan DM A longitudinal study of risk factors for suicide attempts among Operation Enduring Freedom and Operation Iraqi Freedom Veterans. Depress Anxiety. (2018) 35:609–18. 10.1002/da.22736 29637667

[9] Kessler RC Hwang I Hoffmire CA McCarthy JF Petukhova MV Rosellini AJ Developing a practical suicide risk prediction model for targeting high-risk patients in the Veterans health administration. Int J Methods Psychiatr Res. (2017) 26:e1575. 10.1002/mpr.1575 PMC5614864 28675617

[10] Herzog S Tsai J Nichter B Kachadourian L Harpaz-Rotem I Pietrzak RH. Longitudinal courses of suicidal ideation in U.S. military Veterans: a 7-year population-based, prospective cohort study. Psychol Med. (2021) 51:1–10. 10.1017/S0033291721000301 33602367

[11] Sudol K Mann JJ. Biomarkers of suicide attempt behavior: towards a biologic model of risk. Curr Psychiatry Rep. (2017) 19:31. 10.1007/s11920-017-0781-y 28470485

[12] Wilson ST Chesin M Fertuck E Keilp J Brodsky B Mann JJ. Heart rate variability and suicidal behavior. Psychiatry Res. (2016) 240:241–7. 10.1016/j.psychres.2016.04.033 27124209

[13] Bernert RA Kim JS Iwata NG Perlis ML. Sleep disturbances as an evidence-based suicide risk factor. Curr Psychiatry Rep. (2015) 17:15. 10.1007/s11920-015-0554-4 PMC6613558 25698339

[14] Cummins N Scherer S Krajewski J Schnieder S Epps J Quatieri TF. A review of depression and suicide risk assessment using speech analysis. Speech Commun. (2015) 71:10–49. 10.1016/j.specom.2015.03.004

[15] Ryan EP Oquendo MA. Suicide risk assessment and prevention: challenges and opportunities. Focus. (2020) 18:88–99. 10.1176/appi.focus.20200011 33162846 PMC7587888

[16] Wald NJ. The definition of screening. J Med Screen. (2001) 8:1. 10.1136/jms.8.1.1 11373841

[17] Belouali A Gupta S Sourirajan V Yu J Allen N Alaoui A Acoustic and language analysis of speech for suicidal ideation among US veterans. BioData Min. (2021) 14(1):1–17. 10.1186/s13040-021-00245-y 33531048 PMC7856815

[18] Bahraini N Brenner LA Barry C Hostetter T Keusch J Post EP, Assessment of rates of suicide risk screening and prevalence of positive screening results among US veterans after implementation of the veterans affairs suicide risk identification strategy. JAMA Netw Open. 2020;3(10):e2022531-e. 10.1001/jamanetworkopen.2020.22531 33084900 PMC7578771

[19] Kroenke K Spitzer RL Williams JB. The PHQ-9: validity of a brief depression severity measure. J Gen Intern Med. (2001) 16:606–13. 10.1046/j.1525-1497.2001.016009606.x 11556941 PMC1495268

[20] Louzon SA Bossarte R McCarthy JF Katz IR. Does suicidal ideation as measured by the PHQ-9 predict suicide among VA patients? Psychiatry Serv. (2016) 67:517–22. 10.1176/appi.ps.201500149 26766757

[21] Posner K Brown GK Stanley B Brent DA Yershova KV Oquendo MA The Columbia-Suicide Severity Rating Scale: initial validity and internal consistency findings from three multisite studies with adolescence and adults. Am J Psychiatry. (2011) 168:1266–77. 10.1176/appi.ajp.2011.10111704 22193671 PMC3893686

[22] Wortzel HS Homaifar B Matarazzo B Brenner LA. Therapeutic risk management of the suicidal patient: stratifying risk in terms of severity and temporality. J Psychiatr Pract. (2014) 20:63–7. 10.1097/01.pra.0000442940.46328.63 24419312

[23] Simpson SA Goans C Loh R Ryall K Middleton MC Dalton A. Suicidal ideation is insensitive to suicide risk after emergency department discharge: performance characteristics of the Columbia-Suicide Severity Rating Scale Screener. Acad Emerg Med. (2021) 28(6):621–9. 10.1111/acem.14198 33346922

[24] Yarborough BJH Stumbo SP Ahmedani B Rossom R Coleman K Boggs JM Suicide behavior following PHQ-9 screening among individuals with substance use disorders. J Addict Med. (2021) 15(1):55–60. 10.1097/ADM.0000000000000696 32657957 PMC12037181

[25] US Department of Veterans Affairs, US Department of Defense. VA/DoD Clinical Practice Guideline for the assessment and management of patients at risk for suicide. Published May 2019. Available from: https://www.healthquality.va.gov/guidelines/MH/srb/VADoDSuicideRiskFullCPGFinal5088212019.pdf (Accessed June 18, 2022).

[26] Low CA. Harnessing consumer smartphone and wearable sensors for clinical cancer research. npj Digit Med. (2020) 3(140). 10.1038/s41746-020-00351-x PMC7591557 33134557

[27] Jim HSL Hoogland AI Brownstein NC Barata A Dicker AP Knoop H Innovations in research and clinical care using patient-generated health data. CA Cancer J Clin. (2020) 70(3):182–99. 10.3322/caac.21608 32311776 PMC7488179

[28] Tu J Torrente-Rodriguez RM Wang M Gao W. The era of digital health: a review of portable and wearable affinity biosensors. Adv Funct Mater. (2020) 30(29). 10.1002/adfm.201906713

[29] Mandl KD Manrai AK. Potential excessive testing at scale: biomarkers, genomics, and machine learning. JAMA. (2019) 321(8):739–40. 10.1001/jama.2019.0286 30735228 PMC7572222

[30] Kourtis LC Regele OB Wright JM Jones GB. Digital biomarkers for Alzheimer’s disease: the mobile/wearable device opportunity. npj Digit Med. (2019) 2(9). 10.1038/s41746-019-0084-2 PMC6526279 31119198

[31] Pavic M Klaas V Theile G Kraft J Troster G Guckenberger M. Feasibility and usability aspects of continuous remote monitoring of health status in palliative cancer patients using wearables. Oncology. (2020) 98:386–95. 10.1159/000501433 31336377

[32] Asare KO Moshe I Terhorst Y Vega J Hosio S Baumeister H Mood ratings and digital biomarkers from smartphone and wearable data differentiates and predicts depression status: a longitudinal data analysis. Pervasive Mob Comput. (2022) 83:1–13. 10.1016/j.pmcj.2022.101621

[33] Torous J Larsen ME Depp C Cosco TD Barnett I Nock MK Smartphones, sensors, and machine learning to advance real-time prediction and interventions for suicide prevention: a review of current progress and next steps. Curr Psychiatry Rep. (2018) 20(7):51. 10.1007/s11920-018-0914-y 29956120

[34] Sequeira L Perrotta S LaGrassa J Merikangas K Kreindler D Kundur D Mobile and wearable technology for monitoring depressive symptoms in children and adolescents: a scoping review. J Affect Disord. (2020) 265:314–24. 10.1016/j.jad.2019.11.156 32090755

[35] Chiauzzi E Wicks P. Beyond the therapist’s office: merging measurement-based care and digital medicine in the real world. Digit Biomark. (2021) 5:176–82. 10.1159/000517748 34723070 PMC8460973

[36] Onyeaka H Firth J Kessler RC Lovell K Torous J. Use of smartphones, mobile apps and wearables for health promotion by people with anxiety or depression: an analysis of a nationally representative survey data. Psychiatry Res. (2021) 304:114120. 10.1016/j.psychres.2021.114120 34303946

[37] Coravos A Goldsack JC Karlin DR Nebeker C Perakslis E Zimmerman N Digital medicine: a primer on measurement. Digit Biomark. (2019) 3(2):31–71. 10.1159/000500413 32095767 PMC7015383

[38] van den Brink W Bloem R Ananth A Kanagasabapathi T Amelink A Bouwman J Digital resilience biomarkers for personalized health maintenance and disease prevention. Front Digit Health. (2021) 2:54. 10.3389/fdgth.2020.614670 PMC8521930 34713076

[39] Califf RM. Biomarker definitions and their applications. Exp Biol Med. (2018) 243(3):213–21. 10.1177/1535370217750088 PMC5813875 29405771

[40] Sels L Homan S Ries A Santhanam P Scheerer H Colla M SIMON: a digital protocol to monitor and predict suicidal ideation. Front Psychiatry. (2021) 12:890. 10.3389/fpsyt.2021.554811 PMC8280352 34276427

[41] Lemogne C Thomas F Consoli SM Pannier B Jégo B Danchin N. Heart rate and completed suicide: evidence from the IPC cohort study. Psychosom Med. (2011) 73(9):731–6. 10.1097/PSY.0b013e3182365dc7 22021462

[42] Chang C-C Tzeng N-S Kao Y-C Yeh C-B Chang H-A. The relationships of current suicidal ideation with inflammatory markers and heart rate variability in unmedicated patients with major depressive disorder. Psychiatry Res. (2017) 258:449–56. 10.1016/j.psychres.2017.08.076 28886903

[43] Khandoker AH Luthra V Abouallaban Y Hasan MA Chowdhury N Jelinek HF. Reduced QT variability and increased QT/RR slope in ECG signals of depressed patients with suicidal ideation. 2016 Computing in cardiology conference (CinC) (2016). p. 393–6.

[44] Khandoker AH Luthra V Abouallaban Y Saha S Ahmed KI Mostafa R Predicting depressed patients with suicidal ideation from ECG recordings. Med Biol Eng Comput. (2017) 55(5):793–805. 10.1007/s11517-016-1557-y 27538398

[45] Khandoker AH Widatalla N Jelinek H Niizeki K Hadjileontiadis L. Incoherent synchronization between resting state respiratory sinus arrhythmia and respiratory movement in depressed patients with suicidal ideation. In 2018 computing in cardiology conference (CinC) (2018). Vol. 45, p. 1–4.

[46] Woodward SH Khan C Jamison A Arsenault NJ. 0872 Suicidality is associated with elevated sleep heart rate, BMI, and snoring in veterans with chronic severe PTSD. Sleep. (2019) 42:A350–1. 10.1093/sleep/zsz067.870

[47] McCarthy E DeViva JC Southwick SM Pietrzak RH. Self-rated sleep quality predicts incident suicide ideation in US military veterans: results from a 7-year, nationally representative, prospective cohort study. J Sleep Res. (2022) 31(1):e13447. 10.1111/jsr.13447 34328228

[48] Bishop TM Walsh PG Ashrafioun L Lavigne JE Pigeon WR. Sleep, suicide behaviors, and the protective role of sleep medicine. Sleep Med. (2020) 66:264–70. 10.1016/j.sleep.2019.07.016 31727433

[49] Don Richardson J King L St Cyr K Shnaider P Roth ML Ketcheson F Depression and the relationship between sleep disturbances, nightmares, and suicidal ideation in treatment-seeking Canadian armed forces members and veterans. BMC Psychiatry. (2018) 18(1):1–8. 10.1186/s12888-018-1782-z 29921268 PMC6011186

[50] McCarthy MS Hoffmire C Brenner LA Nazem S. Sleep and timing of death by suicide among US veterans 2006–2015: analysis of the American time use survey and the national violent death reporting system. Sleep. (2019) 42(8):zsz094. 10.1093/sleep/zsz094 31180507

[51] Bruce ML. Suicide risk and prevention in Veteran populations. Psychiatr Neurol Aspects oWar. (2010) 1208(1):98–103. 10.1111/j.1749-6632.2010.05697.x 20955331

[52] Na PJ Yaramala SR Kim JA Kim H Goes FS Zandi PP The PHQ-9 item 9 based screening for suicide risk: a validation study of the Patient Health Questionnaire (PHQ)-9 with the Columbia Suicide Severity Rating Scale (C-SSRS). J Affect Disord. (2018) 232:34–40. 10.1016/j.jad.2018.02.045 29477096

[53] Lindh ŠU Dahlin M Beckman K Strömsten L Jokinen J Wiktorsson S A comparison of suicide risk scales in predicting repeat suicide attempt and suicide: a clinical cohort study. J Clin Psychiatry. (2019) 80(6):20485. 10.4088/JCP.18m12707 31747488

[54] Chang BP Tan TM. Suicide screening tools and their association with near-term adverse events in the ED. Am J Emerg Med. (2015) 33:1680–3. 10.1016/j.ajem.2015.08.013 26346049

[55] Runeson B Odeberg J Pettersson A Edbom T Adamsson IJ Waern M. Instruments for the assessment of suicide risk: a systematic review evaluating the certainty of the evidence. PLoS One. (2017) 12(7). 10.1371/journal.pone.0180292 PMC5517300 28723978

[56] Ammerman BA Law KC. Using intensive time sampling methods to capture daily suicidal ideation: a systematic review. J Affect Disord. (2020) 299:108–17. 10.1016/j.jad.2021.10.121 34718039

[57] Walsh CG Johnson KB Ripperger M Sperry S Harris J Clark N Prospective validation of an electronic health record-based, real-time suicide risk model. JAMA Netw Open. (2021) 4(3):e211428. 10.1001/jamanetworkopen.2021.1428 33710291 PMC7955273

[58] Green JD Kearns JC Rosen RC Keane TM Marx BP. Evaluating the effectiveness of safety plans for military Veterans: do safety plans tailored to Veteran characteristics decrease suicide risk? Behav Ther. (2018) 49(6):931–8. 10.1016/j.beth.2017.11.005 30316491

[59] Jacobson NC Bhattacharya S. Digital biomarkers of anxiety disorder symptom changes: personalized deep learning models using smartphone sensors accurately predict anxiety symptoms from ecological momentary assessments. Behav Res Ther. (2022) 149:104013. 10.1016/j.brat.2021.104013 35030442 PMC8858490

[60] Horowitz LM Roaten K Pao M Bridge JA. Suicide prevention in medical settings: the case for universal screening. Gen Hosp Psychiatry. (2020) 63:7–8. 10.1016/j.genhosppsych.2018.11.009 30686522 PMC11500748

[61] Betthauser LM Stearns-Yoder KA McGarity S Smith V Place S Brenner LA. Mobile app for monitoring and clinical outreach in Veterans: mixed methods feasibility and acceptability study. J Med Internet Res. (2020) 22(8. 10.2196/15506 PMC7448171 32779572

[62] Tucker RP Cramer RJ Langhinrichsen-Rohling J Rodriguez-Cue R Rasmussen S Oakey-Frost N Insomnia and suicide risk: a multi-study replication and extension among military and high-risk college student samples. Sleep Med. (2021) 85:94–104. 10.1016/j.sleep.2021.06.032 34298228

[63] Chu C Hom MA Rogers ML Stanley IH Ringer-Moberg FB Podlogar MC Insomnia and suicide-related behaviors: a multi-study investigation of thwarted belongingness as a distinct explanatory factor. J Affect Disord. (2017) 208:153–62. 10.1016/j.jad.2016.08.065 27770645 PMC5154904

[64] Lavigne J Hur K Kane C Au A Bishop T Pigeon W. Comparative safety of sleep prescriptions and suicide attempts in veterans. Value Health. (2018) 21:S203. 10.1016/j.jval.2018.04.1382

[65] Hilberg AM Murphy L Pham S Bernert RA. 0891 Nightmares predict cross-sectional risk for suicidal ideation, but not perceived stigma in a high-risk sample of US military veterans. Sleep. (2019) 42(Suppl. 1):A358-A. 10.1093/sleep/zsz067.889

[66] Capurro D Coghlan S Pires DE. Preventing digital overdiagnosis. JAMA. (2022) 327(6):525–6. 10.1001/jama.2021.22969 35061004

[67] O’Rourke MC Jamil RT Siddiqui W. Suicide Screening and Prevention.

